# Supplementary material for: Genotypic and Phenotypic Diversity of Kluyveromyces marxianus Isolates Obtained from the Elaboration Process of Two Traditional Mexican Alcoholic Beverages Derived from Agave: Pulque and Henequen (Agave fourcroydes) Mezcal
Source: J Fungi (Basel). 2023 Jul 28;9(8):795. doi: 10.3390/jof9080795 (PMC10455534; doi:10.3390/jof9080795)
Supplement: Supplementary file 1 [file jof-09-00795-s001.zip › Table_S1.pdf]

Table S1. Carbon and nitrogen compounds assimilation patterns of the *K. marxianus* isolates obtained from the elaboration process of henequen mezcal and pulque. The two last columns show the results reported for *K. marxianus* in the literature for comparison.

Test	Isolates							Barnett et al. (2000)	Lachance (2011)
	**Kmx11**	**Kmx14**	**Kmx15**	**Kmx16**	**Kmx21**	**Kmx22**	**Kmx24**		
**Assimilation of carbon compounds**									
D-Galactose	+	+	+	+	+	+	+	+	+
L-Sorbose	-	-	-	-	-	-	-	+,-	-
ilusosamine	-	-	-	-	-	-	-	-	-
L Arabinose	-	+	-	+	+	+	+	+,-	v
D-Arabinose	-	-	-	+	-	-	-	-	-
Methyl α-D-glucoside	-	-	-	-	-	-	-	+,-	-
Salicine	+	+	+	+	+	w	+	+,-	v
Arbutin	+	-	+	+	+	+	+	+,-	nd
Melibiose	-	-	-	-	-	-	-	-	-
Melezitose	-	-	-	-	-	-	-	+,-	-
Starch	nd	-	nd	-	-	-	-	+,-	-
Erythritol	-	-	-	-	-	-	-	-	-
D-Glucitol	d	+	+	+	d	w	+	+,-	v
Galactitol	-	-	-	-	-	-	-	-	-
myo-Inositol	-	-	-	-	-	-	-	-	-
2 Keto D gluconate	-	-	-	-	-	-	-	-	-
D-Gluconate	-	-	-	-	-	-	-	-	-
D-Glucuronate	-	-	-	-	-	-	-	-	-
Methanol	-	-	-	-	-	-	-	-	-
**Assimilation of nitrogen compounds**									
Nitrate	-	-	-	-	-	-	-	-	-
Nitrite	-	-	-	-	-	-	-	-	-
Ethylamine	+	+	+	+	+	+	+	+	+

L-Lysine	**+**	**+**	**+**	**+**	**+**	**+**	**+**	**+**	**+**
Cadaverine	**-**	**+**	**+**	**+**	**+**	**+**	**+**	**+**	**+**
Creatine	**-**	**-**	**-**	**-**	**-**	**-**	**-**	**-**	**nd**
Creatinine	**-**	**-**	**-**	**-**	**-**	**-**	**-**	**-**	**nd**

+ positive; **-** negative; **w** weak, d delayed, **v** variable: positive response; **nd** non determined.

Lachance, M.A. Kluyveromyces van Der Walt (1971). In The Yeasts; Kurtzman, C.P., Fell, J.W., Boekhout, T., Eds.; Elsevier: Amsterdam, The Netherlands, 2011; Volume 2, pp. 471–481, ISBN 9780444521491.

Barnett, J.A.; Payne, R.W.; Yarrow, D.; Barnett, L. Yeasts: Characteristics and Identification; Cambridge University Press: Cambridge, UK, 2000; ISBN 9780521573962.
